# Orbital Tumours in Northern Malaysia: A Five-Year Review

**DOI:** 10.7759/cureus.20941

**Published:** 2022-01-04

**Authors:** Tan Qi-Xian, Tan Chew-Ean, Adlina Abdul Rahim, Rona A Nasaruddin

**Affiliations:** 1 Ophthalmology, Hospital Sultanah Bahiyah, Alor Setar, MYS; 2 Ophthalmology, Universiti Kebangsaan Malaysia Medical Centre, Kuala Lumpur, MYS

**Keywords:** five years, biopsy, histopathological diagnoses, northern malaysia, orbital tumours

## Abstract

Background: This study was conducted with the objective to examine demographic statistics and histopathological diagnoses of orbital biopsies from cases referred to the Oculoplastic subspecialty of the Ophthalmology department in Hospital Sultanah Bahiyah, Kedah, from 2016 to 2020.

Method: This study is a case series of 28 patients who underwent orbital biopsy.

Result: A total of 34 orbital biopsies from 28 patients were recorded. The mean age was 48.3 ± 19.1 years old. 22 (78.4%) cases manifested unilaterally and six (21.4%) manifested bilaterally. The commonest presentations were orbital mass (36.6%) and proptosis (24.4%). The mean duration of the presentation was 16.2 ± 19.5 months. Fourteen (50.0%) patients underwent orbital biopsy within six months of symptoms. 52.8% of the tumours are situated at supero-temporal region of the orbit. 53.0% (18) situated in extraconal space of orbit. Out of all, 23 (67.6%) cases were benign and 11 (32.4%) cases were malignant. All were primary in origin (100%). The commonest orbital tumours reported were malignant lymphoma (29.4%), reactive lymphoid hyperplasia (14.7%), non-caseating granulomatous inflammation (11.8%), non-granulomatous inflammation (5.9%) and cavernous haemangioma (5.9%). Of all 10 malignant lymphomas from eight patients (two were bilateral eyes) were all mucosa-associated lymphoid tissue (MALT) of primary non-Hodgkin lymphomas. None of the cases were reported to be metastasis from the systemic spread. The majority of patients' eyes (20, 58.8%) remained the same visual acuity post-biopsy while six (17.6%) eyes had improvement in visual acuity and eight (23.5%) eyes had reduced postoperative visual acuity.

Conclusion: Malignant tumours are more common in the elder age group especially malignant lymphoma which is in contrast to Caucasian populations. Understanding the relative incidence of these various orbital tumours is essential to patient evaluation and management.

## Introduction

The most frequently encountered primary orbital tumours in adults are lymphoproliferative lesions (8.0%-24.0%) [1-3] and cavernous malformations (9.5%-17.0%) [3-5]. In children, the most common orbital tumours tend to be benign including cystic (6.0-46.0%) [2-7] and vascular lesions (6.0%-31.0%) [2-7]. There is a broad spectrum of orbital tumours based on demographics, site of origin, anatomical location and histopathological features. Orbital tumours with unvaried presentations can yield distinctive outcomes and hence it poses dilemmas and challenges in terms of diagnosis, imaging and treatment.

To our best knowledge, there was limited data available in the literature which specifically described the epidemiology and histopathological diagnoses of orbital tumours in Malaysia. This study aimed to determine demographic statistics and histopathological diagnoses of orbital biopsies in the oculoplastic centre in northern Malaysia.

## Materials and methods

Methodology

This is a case series study.

Inclusion criteria: Patients diagnosed with orbital tumours and underwent eyelid tumour biopsy in Hospital Sultanah Bahiyah, Kedah between January 2016 and December 2020.

Exclusion criteria: Patient, who defaulted follow up during treatment period, has incomplete clerking history and examination or has orbital tumours however refused for surgery.

Data collection: Patients with orbital lesions from district hospitals in Northern Malaysia were referred to the oculoplastic centre Hospital Sultanah Bahiyah (HSB). All patients were examined and radiological imaging was performed. Orbital biopsies were performed and sent for histopathological diagnosis. Data such as demographic (age, gender, race), clinical presentation, orbital lesion topography and histopathological diagnoses were collected. The results were analysed by using SPSS version 22.0 (SPSS Inc., Chicago, IL, USA). This study obtained ethical approval (National Medical Research Registry NMRR ID-21-02292-4Y3) and was conducted according to the Declaration of Helsinki principles. Written informed consent was obtained from all the patients before the orbital biopsy.

## Results

A total of 34 orbital biopsies from 28 patients were identified during the five years study period. The mean age was 48.3 ± 19.1 years old. The range of age was from six to 76 years old. The most frequent age group in this study was between 19 and 64 years old (22, 78.6%). Nineteen (67.9%) were male and nine (32.1%) were female, 24 (85.7%) were Malay race, three (10.7%) were Chinese race and one (3.6%) was Indian race. 22 (78.6%) cases of orbital tumours manifested unilaterally and six (21.4%) manifested bilaterally. Out of 34 orbital biopsies, 23 (67.6%) cases were benign and 11 (32.4%) cases were malignant. All these 10 malignant cases were primary in origin (100%). The demographic factors of all orbital biopsies are summarised in Table 1.

**Table 1:** Demographic factor details of all orbital biopsies in HSB (n = 28) HSB - Hospital Sultanah Bahiyah Total % is more than100% because of rounding

Demographic factors	Frequency, n (%)
Age group	
<18	1 (3.6)
19-64	22 (78.6)
>65	5 (17.9)
Gender	
Male	19 (67.9)
Female	9 (32.1)
Race	
Malay	24 (85.7)
Chinese	3 (10.7)
Indian	1 (3.6)
Laterality	
Unilateral	22 (78.6)
Bilateral	6 (21.4)

The mean duration of presentation in patients with orbital tumours was 16.2 ± 19.5 months. Fourteen (50.0%) patients underwent orbital biopsy within six months of symptoms and 12 (42.9%) patients underwent orbital biopsy after one year of symptoms. Fifteen (36.6%) patients presented with complaints of mass or swelling in the orbital region. This was followed by proptosis in 10 (24.4%), blurred vision in five (12.2%), ptosis in four (9.8%), diplopia in four (9.8%) and eye redness in three (7.3%).

In 34 eyes of 28 patients, 13 (38.2%) eyes had visual acuity of 6/9 or better prior to surgery, 10 (29.4%) eyes had visual acuity of 6/12 to 6/18, 8 (23.6%) eyes in between 6/21 to 6/60. Three (8.8%) eyes had visual acuity worse than 3/60. Out of 34 tumours that underwent biopsy, 19 (52.8%) were located in supero-temporal region of the orbit, nine (26.5%) cases were located in intraconal space, 18 (53.0%) cases in extraconal space and seven (20.5%) cases were both in extraconal and intraconal space (Table 2).

**Table 2:** Clinical presentations of 28 patients who underwent orbital tumour biopsies in HSB (n = 34) HSB - Hospital Sultanah Bahiyah Total % is more than 100% because of rounding.

Clinical Presentations	Frequency, n (%)
Initial Presentations	
Mass	15 (36.6)
Proptosis	10 (24.4)
Blurred vision	5 (12.2)
Ptosis	4 (9.8)
Diplopia	4 (9.8)
Red eye	3 (7.3)
Visual Acuity based on Snellen Chart preoperatively	
6/9 or better	13 (38.2)
6/12 to 6/18	10 (29.4)
6/21 to 6/60	8 (23.6)
Worse then 6/60	3 (8.8)
Visual Acuity based on Snellen Chart postoperatively	
6/9 or better	9 (26.5)
6/12 to 6/18	9 (26.5)
6/21 to 6/60	12 (35.3)
Worse then 3/60	4 (11.8)
Tumour location (sector)	
Superotemporal	19 (52.8)
Inferonasal	5 (13.9)
Superior	2 (5.6)
Nasal	2 (5.6)
Inferior	3 (8.3)
Temporal	2 (5.6)
Diffuse	3 (8.3)
Relation to the conal space	
Intraconal	9 (26.5)
Extraconal	18 (53.0)
Extraconal and intraconal	7 (20.5)

Out of 34 orbital tumours biopsied in HSB, the commonest orbital tumours were malignant lymphoma (29.4%), followed by inflammatory lesions such as reactive lymphoid hyperplasia (14.7%) and non-caseating granulomatous inflammation (11.8%). There were two out of three cases of benign lymphoid tissues that involved bilateral eyes. The histopathological diagnoses of 34 tumours biopsied in 28 eyes in HSB are depicted in Table 3. 

**Table 3:** The histopathological diagnoses of tumour biopsies in HSB (n = 34) HSB - Hospital Sultanah Bahiyah Total % is more than 100% because of rounding.

Types of Tumours	Frequency, n (%)
Cystic	2 (5.9)
Dermoid Cyst	1 (2.9)
Hidrocystoma	1 (2.9)
Vasculogenic	6 (17.6)
Cavernous Haemangioma	2 (5.9)
Capillary Haemangioma	1 (2.9)
Lymphangioma	1 (2.9)
Venous Lymphatic Malformation	1 (2.9)
Haemangioma, Non-specific	1 (2.9)
Neural	1 (2.9)
Schwannoma	1 (2.9)
Mesenchymal	1 (2.9)
Solitary Fibrous Tumour	1 (2.9)
Lacrimal gland	2 (5.9%)
Pleomorphic Adenoma	1 (2.9)
Adenoid Cystic Carcinoma	1 (2.9)
Lymphoid/Leukaemia	16 (47.1)
Malignant Lymphoma	10 (29.4)
Reactive Lymphoid Hyperplasia	5 (14.7)
Plasmacytoma	1 (2.9)
Inflammatory	6 (17.6)
Non-Caseating Granulomatous Inflammation	4 (11.8)
Non-Granulomatous Inflammation	2 (5.9)

## Discussion

There were not many epidemiology and histopathological diagnoses of orbital tumours related studies published locally. Hence there were limited references to compare with our studies due to the multi-racial community in Malaysia.

In this study, the mean age of patients (48.3 ± 19.1 years) was higher compared to the studies by Liza et al., which was 43.9 ± 21.7 years [8]. We also reported higher male preponderance (male: female = 67.9%: 32.1%) rather than females preponderance in previous study in Malaysia (male: female = 47.5%: 52.5%) [8] and overseas where females were predominant (53.3%-57.0%) [2,3,5]. This dissimilarity can be due to the low sample size in our study. Most patients presented with the mean duration of follow-up post-surgery of all patients were 23.2 ± 20.4 months. The majority of patients' eyes (20, 58.8%) remained the same visual acuity post-operatively while six (17.6%) eyes had improvement in visual acuity and eight (23.5%) eyes had reduced postoperative visual acuity. Five (14.7%) patients developed cataracts upon follow up, one (2.9%) patient had changes of refractive error post operation which showed myopic shift [9], one (2.9%) patient had advanced progression of normotensive glaucoma, and one (2.9%) patient underwent evisceration. The progression of cataracts was likely due to the advancing age of the patients as there were no previous studies indicating cataract was the direct sequence of orbital biopsy. Our study did not observe any immediate visual loss following orbital biopsy.

In our study, the most common benign orbital tumours were vasculogenic (17.6%) and inflammatory lesions (17.6%). This is almost similar to the study by Henderson et al. [4] where 15.0% were vasculogenic lesions and 8.0% were inflammatory lesions. Inversely, in another study by Kennedy et al. [10], 9% were vasculogenic lesions and 16% were inflammatory lesions. These discrepancies can be due to racial aberration in our studies. The average age of patients with an inflammatory lesion (40.2 years) was higher than patients with vasculogenic lesions (31.3 years). In contrast, a previous study stated that the occurrence of vasculogenic orbital masses was elder age group (41-48 years) [11].

The most common malignant orbital tumours in our study were malignant lymphoma (29.4%). The frequency is higher than reported in a previous study (12.5%) [8]. Malignant lymphoma was seen in elder age at an average age of 64 years old, with ages ranging from 53 to 76 years. The clinical characteristics are diverse according to the location of tumours similar to previously reported [12], and we also observed multiple symptoms including conjunctival injection, lid swelling, proptosis, painless palpable mass, ptosis, and blurred vision. Of all 10 malignant lymphomas from eight patients (two were bilateral eyes) were all mucosa-associated lymphoid tissue (MALT) of primary non-Hodgkin lymphomas. One patient had distant metastasis to the lung which was detected through computerised tomography of thorax imaging after diagnosis of orbital lymphoma made. Seven patients were referred to a haematologist for chemotherapy treatment. None of the eight patients had a recurrence of growth upon follow-up. In 2007, Liza-Sharmini et al. [8] reported two out of five cases (5.0%) in the lacrimal gland and three cases in orbital lymphoid tissue (7.5%). While in our current study, none of the malignant lymphomas were biopsied from the lacrimal gland. Both the incidence rates of malignant lymphoma and reactive lymphoid hyperplasia in our study were almost comparable to the Japanese population, [2] where malignant lymphoma was 23.7% and reactive lymphoid hyperplasia was 18.4%. The correlative incidence rates of malignant lymphoma were also reported by Demirci et al. (24.0%) [1] and Ting et al. (24.7%) [13]. On the other hand, malignant lymphoma was reported to be lower incidence in the Caucasian population, ranging from 8% to 13% [4,5,10].

In the present study, one (2.9%) basaloid subtype of adenoid cystic carcinoma of the lacrimal gland presenting as a space-occupying orbital lesion in the intraconal and extraconal region. It turned out to be a secondary tumour from the lacrimal gland after radiological imaging and histopathological examination confirmation. The patient subsequently underwent subtotal exenteration.

None of the cases were reported to be metastasis from the systemic spread. Rhabdomyosarcoma, primary melanocytic lesion, optic nerve and meningioma-related lesions were not found in our study. Retinoblastoma cases were not referred to Oculoplastic subspecialty in our hospital hence excluded. Thyroid orbitopathy cases were diagnosed radiologically and biopsy was not routinely done in these cases. Table 4 showed a published series of relative frequencies of orbital tumours in the general population in various countries.

**Table 4:** Published series of relative frequencies of orbital tumours in the general population in various countries. NA: Not applicable Total % is more than 100% because of rounding.

Classification	Our study, Malaysia	Henderson, USA [4]	Shields, USA [5]	Kennedy, USA [10]	Ohtsuka, Japan [2]	Al- Mamoori, Iraq [14]	Liza- Sharmini, Malaysia [8]
Thyroid Orbitopathy	NA	NA	NA	6	NA	NA	10
Cystic Lesions	2	12	30	11	5	30	18
Vasculogenic Lesions	6	15	6	9	13	12	8
Peripheral Nerves Tumours	1	4	2	4	4	NA	NA
Optic Nerve and Meningeal Tumours	NA	11	2	5	4	4	5
Fibrocytic Tumours	NA	13	2	<1	1	3	NA
Osseous and Fibro-Osseous	NA	3	2	3	1	4	NA
Cartilageous Lesions	NA	<1	<1	NA	<1	NA	NA
Mesenchymal and Adipose Lesions	1	<1	4	<1	1	2	NA
Rhabdomyosarcoma	NA	2	1	1	1	1	NA
Lacrimal Gland Lesions	2	6	13	5	11	2	15
Primary Melanocytic Tumours	NA	<1	<1	NA	NA	1	NA
Metastatic Lesions	NA	2	3	3	3	4	3
Lymphoid Tumours and Leukaemia	16	8	10	13	42	1	8
Secondary Tumours	1	23	11	6	10	16	18
Histiocytic Lesions	NA	1	<1	<1	NA	NA	3
Inflammatory Lesions	6	8	13	16	NA	15	15
Unclassified Lesions	NA	NA	2	2	<1	2	NA
Normal	NA	NA	<1	<1	NA	NA	NA
Other	NA	NA	NA	15	1	1	NA

## Conclusions

Malignant tumours are more common in the elder age group, especially malignant lymphoma. Understanding the relative incidence of these various orbital tumours is essential to patient evaluation and management. Systemic evaluations and follow-ups are essential in metastasis and recurrence surveillance. The greatest challenge was the numbers in our study may possibly under-reported. Numerous of our local patients refused surgical intervention for suspicious lesions at our centre in view of poor health education and belief in traditional medications. There are several limitations to our study such as incomplete medical records and lack of socio-demographic information in view of the retrospective nature of this study.
